# Optoelectronic characteristics of YAG phosphor-incorporated ZnO films deposited by ultrasonic spray pyrolysis

**DOI:** 10.1186/1556-276X-7-627

**Published:** 2012-11-15

**Authors:** Lung-Chien Chen, Chien-Chung Huang

**Affiliations:** 1 Department of Electro-optical Engineering, National Taipei University of Technology, 1, sec.3, Chung-Hsiao E. Road, Taipei, 106, Taiwan

**Keywords:** YAG, ZnO, Photoluminescence, Nanoflower

## Abstract

This work presents a novel white light device. An yttrium aluminum garnet (YAG) phosphor-incorporated zinc oxide (ZnO) film is deposited on a slide glass substrate by ultrasonic spray pyrolysis. A nanoflower consisting of a hexagonal nanopetal is formed on the surfaces of the samples, and the sizes of the nanopetal are approximately 200 to 700 nm. Additionally, the nanopetal becomes blunted with an increasing incorporated amount of YAG. As the incorporated amount is 1.5 and 2.5 wt.%, the photoluminescence color of the YAG-incorporated ZnO film is nearly white, possibly contributing to the YAG emission and the band-to-deep level transition in the ZnO film.

## Background

Solid state lighting technology is the next generation light source, owing to its potential luminescence efficiency. GaN-based white light-emitting diode (LED) is the most widely used solid state light source because of its long lifetime, high energy efficiency, small size, ability to produce color light directly without filtering, and integration with other semiconductor electronic elements [1-6]. However, prohibitively high manufacturing costs, unstable production in run-to-run and wafer-to-wafer, inadequate light uniformity, and inferior color rendering index represent major obstacles to GaN-based LED as a main light source in the future. The first two obstacles are attributed to the expensive growth technology of metal-organic chemical vapor deposition and the Ga-N bonding mechanism. The latter two obstacles are caused by a mixture of blue and yellow lights. White LED consists of a blue LED chip and a yellow phosphor coating. A non-conformal phosphor coating causes thickness of phosphor coating layer variation and inadequate color uniformity.

Therefore, this study describes a novel white light device for light source. ZnO is commonly used as a material for optical device applications in the UV range owing to its wide direct bandgap (3.37 eV) [7-10]. An n-ZnO/p-GaN heterostructure LED was reported [11]. However, that is a UV range structure, and no any optical characteristics were demonstrated. In this work, an yttrium aluminum garnet (YAG) phosphor-incorporated ZnO film is deposited by ultrasonic spray pyrolysis. The optoelectronic characteristics are also studied. Additionally, the crystallinity of YAG phosphor-incorporated into the ZnO films is studied using X-ray diffraction (XRD) analysis.

## Methods

YAG phosphor-incorporated ZnO film was deposited by ultrasonic spray pyrolysis on slide glass substrates at atmospheric pressure in nitrogen (N_2_) gas, at a flow rate of 100 sccm for 30 min. The YAG phosphor-incorporated ZnO film (YAG phosphor at 0, 0.5, 1.5, and 2.5 wt.%; NYAG4156 phosphor, INTEMATIX, Fremont, CA, USA) was produced by spraying aqueous solutions. The spraying aqueous solution preparation is as follows: (1) a solution consisted of Zn(CH_3_COO)_2_·2H_2_O (0.2 M) and CH_3_COONH_4_·2H_2_O (0.2 M) with 1:3 proportional ratio which were used as sources of ZnO, and (2) the NYAG4156 phosphor powder was added into the ZnO source solution to form the spraying aqueous solution, where the NYAG4156 powder is suspendible. A slide glass was used as the substrate, which was etched with HCl for 5 min before deposition. An aerosol of the precursor solution was then generated using a commercial ultrasonic nebulizer. Next, the morphology of the film was studied by field-emission scanning electron microscope (FESEM). The resistivity and the mobility of the film were studied by Hall measurement. The crystallinity was investigated by XRD using a rotating anode Rigaku X-ray diffractometer (Mac Science Corporation, Yokohama, Japan) with Cu-Kα_1_ radiation at a wavelength of 1.54 Å, where the radiation was generated at 45 kV and 40 mA. Notably, the film had a polycrystalline structure. Additionally, photoluminescence (PL) was measured at room temperature (RT). The excitation source for photoluminescence was a frequency-quadrupled Nd:YAG laser, which emitted 266 nm, 6 ns pulses at a 5-Hz repetition rate.

## Results and discussion

**Figure 1:**
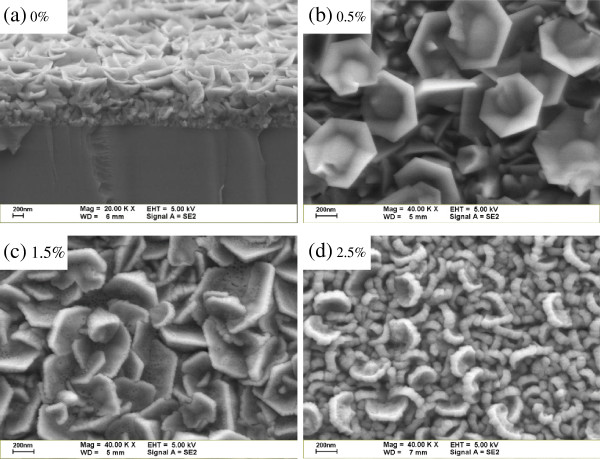
**FESEM micrographs.** YAG-incorporated ZnO films with various incorporated amounts: (**a**) 0%, (**b**) 0.5%, (**c**) 1.5%, and (**d**) 2.5%.

Figure 1 shows the FESEM micrographs of the YAG phosphor-incorporated ZnO films with various incorporated amounts. Following a close examination of the cross-sectional and top-view images of the samples, the micrographs indicate that the nanoflower consists of hexagonal nanopetal on the surface of the film. No nanorod and nanofiber structures were observed as other published articles [12,13]. The nanopetal sizes were approximately 200 to 700 nm. The nanopetal becomes blunted with an increasing incorporated amount of YAG from 0.5% to 2.5%. The blunted nanopetal might be responsible for the degradation of the nanocrystalline ZnO grains because the lattice constant of the YAG is larger than that of the ZnO [14,15]. Despite the attention paid to the ZnO nanoflower in the literature, the nanoflower and nanopetal processes still remain unclear [10,16-19]. However, the origin of the hexagonal nanoflower may contribute to the decomposition and random nucleation of the solution precursor leading to the formation of the three-dimensional ZnO nuclei [10]. As the growth process is terminated, the three-dimensional growth becomes two-dimensional owing to the reduction of the source and the aggregation of the residue precursor, subsequently leading to the formation of the hexagonal nanopetal on the surface of the sample.

**Figure 2:**
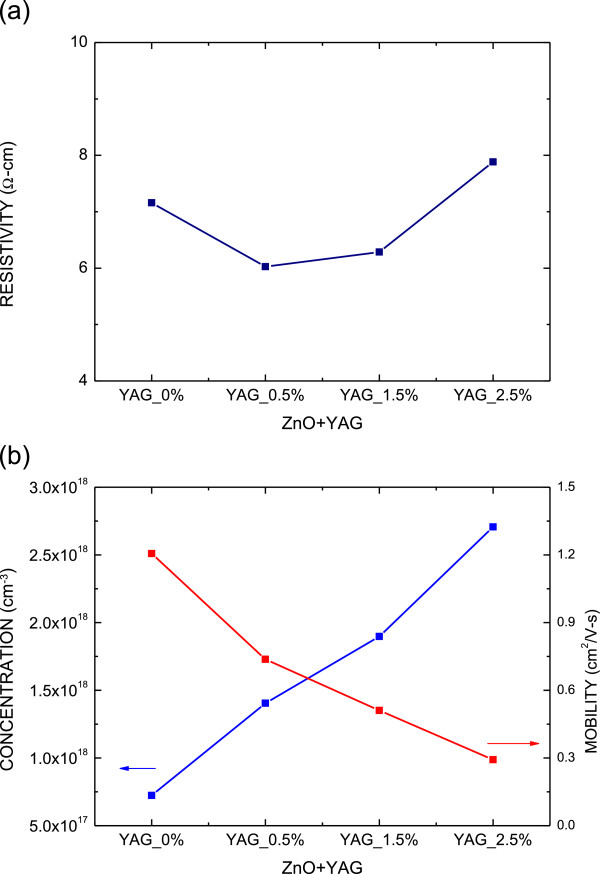
**Resistivity, carrier concentration, and mobility.** (**a**) The resistivity as a function of the incorporated amount of YAG phosphor. (**b**) The carrier concentration and the mobility as a function of the incorporated amount of YAG phosphor.

Figure 2a shows the resistivity as a function of the incorporated amount of YAG phosphor. The resistivity is nearly constant, ranging from 6 to 8 Ω cm. Figure 2b plots both the carrier concentration and the mobility as a function of the incorporated amount of YAG phosphor. As the incorporated amount of YAG phosphor increases to 2.5 wt.%, the carrier concentration likely increases to 2.7 × 10^18^ cm^−3^, and simultaneously, the mobility decreases to 0.3 cm^2^/Vs. The decrease of mobility and the increase of carrier concentration, as shown in Figure 2b, may be attributed to the degradation of the ZnO film crystallinity and the increasing defects caused by the incorporation of the YAG phosphor.

**Figure 3:**
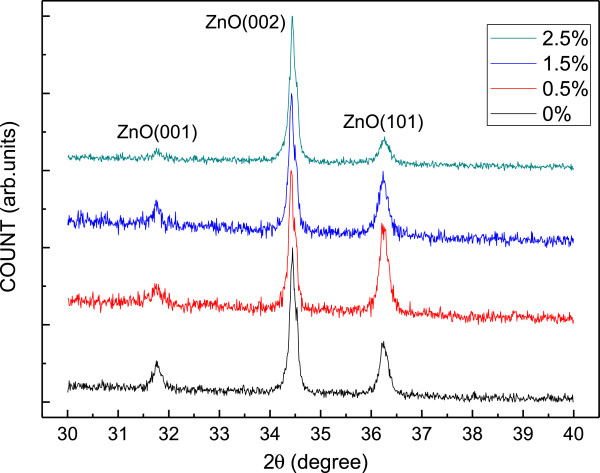
X-ray diffraction patterns of the ZnO films with various incorporated amounts of YAG phosphor.

Figure 3 shows a typical XRD pattern of the YAG phosphor-incorporated ZnO film deposited on a sapphire substrate prepared by the ultrasonic spraying pyrolysis method. The XRD patterns are normalized to the same main peak intensity. Three dominant diffraction peaks, i.e., ZnO(100) (2*θ* = 31.76°), ZnO(002) (2*θ* = 34.46°), and ZnO(101) (2*θ* = 36.27°), are observed. Experimental results indicate that the lattice parameters are *a* = 3.244 Å and *c* = 5.199 Å. The film demonstrates a polycrystalline structure. The sample with YAG of 0.5 wt.% has the maximum ZnO(101) diffraction peak height. As the incorporated amount of YAG phosphor increases more than 1.5 wt.%, the intensity in ZnO(100) and ZnO(101) diffraction peaks decreases. A slight shift in the XRD patterns were observed from 34.445° to 34.425° when the YAG phosphor was incorporated into the ZnO films. This may be attributed to the lattice constant of incorporated YAG larger than that of the ZnO [14,15].

**Figure 4:**
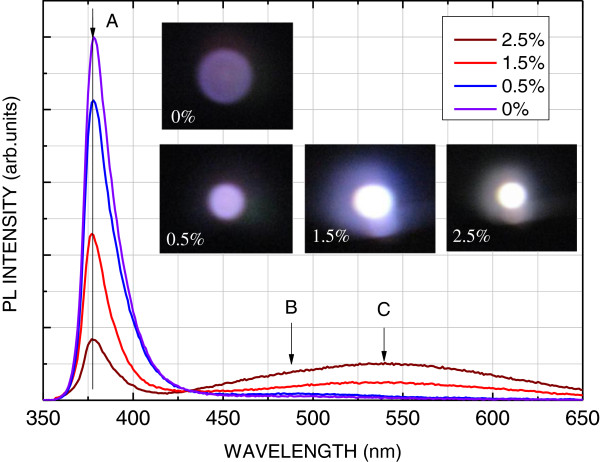
**RT PL spectra of ZnO films with various incorporated amounts of YAG phosphor.** The inset shows the photoexcited luminescent photographs,

Figure 4 presents the RT PL spectra of the ZnO films with various incorporated amounts of YAG phosphor. The inset shows the photoexcited luminescent photographs. According to Figure 4, the RT PL spectrum of the ZnO film without a YAG phosphor reveals one peak, denoted as peak A, i.e., at approximately 3.28 eV (378 nm) and a broad weak band denoted as peak B at approximately 2.557 eV (485 nm). Peak A has the shortest wavelength and, therefore, is interpreted as being associated with free-exciton or band-to-band recombination in the ZnO. Additionally, its position is reasonably close to that of the bandgap of ZnO at RT, which is approximately 3.285 eV (377.5 nm) [7-9,20]. Peak B may be attributed to the band-to-deep level transition in the ZnO film. As the YAG phosphor incorporated into the ZnO films is more than 1.5 wt.%, peak C emerges at around 540 nm. Peak C is associated with the emission of the YAG phosphor. As the incorporated amount is 1.5 and 2.5 wt.%, the color of photoluminescence is nearly white, as shown in Figure 4. The white light may contribute to the wide emission band ranging from 420 to 650 nm, as merged by peaks B and C.

## Conclusion

In summary, an YAG phosphor-incorporated ZnO film is deposited on a slide glass substrate by ultrasonic spray pyrolysis. A nanoflower consisting of a hexagonal nanopetal is formed on the surface of the samples; in addition, the sizes of the nanopetal are approximately 200 to 700 nm. The origin of the hexagonal nanoflower may contribute to the three-dimensional growth becoming two-dimensional, owing to the reduction of the source and aggregation of the residue precursor as the growth process is terminated. Consequently, the hexagonal nanopetal is formed on the surface of the sample. This study also examines the PL spectra of the samples. As the incorporated amount of the YAG is 1.5 and 2.5 wt.%, the photoluminescence color of the YAG-incorporated ZnO films is nearly white. This color may contribute to the wide emission band ranging from 420 to 650 nm, as caused by the YAG emission and the band-to-deep level transition in the ZnO film.

## Competing interests

The authors declare that they have no competing interests.

## Authors' contributions

LCC wrote the paper, designed the experiments, and analyzed the data. CCH grew the samples and did all the measurements. Both authors read and approved the final manuscript.

## References

[B1] Shen YC Wierer JJ Krames MR Ludowise MJ Misra MS Ahmed F Kim AY Mueller GO Bhat JC Stockman SA Martin PS Optical cavity effects in InGaN/GaN quantum-well-heterostructure flip-chip light-emitting diodes Appl Phys Lett 2003 82 2221 10.1063/1.1566098

[B2] Mukai T Yamada M Nakamura S Characteristics of InGaN-based UV/blue/green/amber/red light-emitting diodes Jpn J Appl Phys 1999 38 3976 3981 10.1143/JJAP.38.3976

[B3] Chen LC Huang YL High reliability GaN-based light-emitting diodes with photo-enhanced wet etching Solid State Electron 2004 48 1239 1242 10.1016/j.sse.2004.02.003

[B4] Chen LC Huang JB Cheng PJ Hong LS InGaN blue light-emitting diodes with ZnO nucleation layers prepared by the sol–gel method Semicond Sci Technol 2007 22 1178 1182 10.1088/0268-1242/22/10/017

[B5] Chen LC Ho YM Ag and zinc oxide doped indium oxide ohmic contacts to p-type GaN for filp-chip LED applications J Phys D: Appl Phys 2007 40 6514 6517 10.1088/0022-3727/40/21/007

[B6] Chen LC Tien CH Mu CS Effects of spin-polarized injection and photo-ionization of MnZnO film on GaN-based light-emitting diodes Opt Express 2010 18 2302 2308 10.1364/OE.18.002302 20174059

[B7] Zhang XH Chua SJ Yong AM Yang HY Lau SP Yu SF Sun XW Miao L Tanemura M Tanemura S Exciton radiative lifetime in ZnO nanorods fabricated by vapor phase transport method Appl Phys Lett 2007 90 013107 10.1063/1.2429019

[B8] Danhara Y Hirai T Harada Y Ohno N Exciton luminescence of ZnO fine particles Phys Stat Sol (c) 2006 3 3565 3568 10.1002/pssc.200672110

[B9] Lim JH Kang CK Kim KK Park IK Hwang DK Park SJ UV electroluminescence emission from ZnO light-emitting diodes grown by high-temperature radiofrequency sputtering Adv Mater 2006 18 2720 2724 10.1002/adma.200502633

[B10] Su SC Lu YM Zhang ZZ Shan CX Yao B Li BH Shen DZ Zhang JY Zhao DX Fan XW The optical properties of ZnO/ZnMgO single quantum well grown by P-MBE Appl Surf Sci 2008 254 7303 7305 10.1016/j.apsusc.2008.05.329

[B11] Li WC Tsai HL Chen HC Wu MK Chen HR Chen MJ Yang JR Shiojiri M Structural investigation of n-ZnO/p-GaN ultraviolet light-emitting diodes grown by atomic layer deposition Funct Mater Lett 2011 4 221 224 10.1142/S1793604711002044

[B12] Dhara S Giri PK Rapid thermal annealing induced enhanced band-edge emission from ZnO nanowires, nanorods and nanoribbons Funct Mater Lett 2011 4 25 29 10.1142/S1793604711001658

[B13] Esmaeilpour Ganji M Bazargan AM Keyanpour-Rad M Bahrevar MA Morphological and optical characterization of electrospun zinc oxide nanofibers Funct Mater Lett 2011 3 141 145

[B14] Roh HS Kim DH Park IJ Song HJ Hur SH Kim DW Hong KS Template-free synthesis of monodispersed Y_3_Al_5_O_12_:Ce^3+^ nanosphere phosphor J Mater Chem 2012 22 12275 12280 10.1039/c2jm30598d

[B15] Xu YN Ching WY Electronic structure of yttrium aluminum garnet(Y3Al5O12) Phys Rev B 1999 59 10530 10535 10.1103/PhysRevB.59.10530

[B16] Suh HW Kim GY Jung YS Choi WK Byun DJ Growth and properties of ZnO nanoblade and nanoflower prepared by ultrasonic pyrolysis J Appl Phys 2005 97 044305 10.1063/1.1849825

[B17] Zhang N Yi R Shi RR Gao GH Chen G Liu XH Novel rose-like ZnO nanoflowers synthesized by chemical vapor deposition Mater Lett 2009 63 496 499 10.1016/j.matlet.2008.11.046

[B18] Prabakar K Son MK Kim WY Kim HJ TiO2 thin film encapsulated ZnO nanorod and nanoflower dye sensitized solar cells Mater Chem Phys 2011 125 12 14 10.1016/j.matchemphys.2010.09.028

[B19] Ardakani AG Pazoki M Mahdavi SM Bahrampour AR Taghavinia N Ultraviolet photodetectors based on ZnO sheets: the effect of sheet size on photoresponse properties Appl Surf Sci 2012 258 5405 5411 10.1016/j.apsusc.2012.02.024

[B20] Wang XH Yao B Shen DZ Zhang ZZ Li BH Wei ZP Lu YM Zhao DX Zhang JY Fan XW Guan LX Cong CX Optical properties of p-type ZnO doped by lithium and nitrogen Solid State Commum 2007 141 600 604 10.1016/j.ssc.2007.01.002

